# Differences in speciation progress in feather mites (Analgoidea) inhabiting the same host: the case of *Zachvatkinia* and *Alloptes* living on arctic and long-tailed skuas

**DOI:** 10.1007/s10493-014-9856-1

**Published:** 2014-10-24

**Authors:** Miroslawa Dabert, Stephen J. Coulson, Dariusz J. Gwiazdowicz, Børge Moe, Sveinn Are Hanssen, Elisabeth M. Biersma, Hanne E. Pilskog, Jacek Dabert

**Affiliations:** 1 Molecular Biology Techniques Laboratory, Faculty of Biology, Adam Mickiewicz University, Umultowska 89, 61-614 Poznan, Poland; 2 Department of Arctic Biology, University Centre in Svalbard, P.O. Box 156, 9171 Longyearbyen, Norway; 3 Department of Forest Protection, Poznan University of Life Sciences, Wojska Polskiego 71c, 60-625 Poznan, Poland; 4 Norwegian Institute for Nature Research, P.O. Box 5685, 7485 Sluppen, Trondheim, Norway; 5 Arctic Ecology Department, Fram Centre, Norwegian Institute for Nature Research, 9296 Tromsø, Norway; 6 Arctic Centre, University of Groningen, P.O. Box 716, 9700 AS Groningen, The Netherlands; 7 British Antarctic Survey, High Cross, Madingley Road, Cambridge, CB3 0ET UK; 8 Department of Animal Morphology, Institute of Environmental Biology, Adam Mickiewicz University, Umultowska 89, 61-614 Poznan, Poland

**Keywords:** DNA barcoding, Haplotype network, Coalescence, Species delimitation, Spitsbergen, Ectocommensal dispersion, *Stercorarius*

## Abstract

Recent molecular phylogenetic analyses have revealed that some apparently oligoxenous feather mite species are in fact monoxenous cryptic species with little morphological differentiation. In this study we analyzed two species, *Zachvatkinia isolata* (Avenzoariidae) and *Alloptes* (*Sternalloptes*) *stercorarii* (Alloptidae) which prefer different parts of the plumage of two sister species of birds: arctic skua (*Stercorarius parasiticus*) and long-tailed skua (*S. longicaudus*) breeding on tundra in the High Arctic archipelago of Svalbard. Given that there are no reports about hybridization events between the host species, we expected that both skuas would have a species-specific acarofauna. The genetic distances among DNA-barcode sequences (COI and 28S rDNA), phylogenetic tree topologies, and haplotype networks of the COI sequences of mites suggested extensive gene flow in *Z. isolata* between and within populations inhabiting both skua species, whereas the *Alloptes* populations were host specific and sufficiently genetically separated as to warrant species-level status. The discrepancy in the genetic structure of *Alloptes* and *Zachvatkinia* populations suggests frequent but transient contacts between the two skua species in which the probability of mite exchange is much higher for *Zachvatkinia*, which is present in high numbers and inhabits exposed parts of primary flight feathers, than for the less abundant *Alloptes* that lives primarily in more protected and inaccessible parts of the plumage. We discuss the possible nature of these contacts between host species and the area(s) where they might take place. The star-like structures in the haplotype network as well as high haplotype diversity and low nucleotide diversity observed in *Z. isolata* are concordant with the known dispersal strategy of feather mites: vertical colonization of new host individuals followed by rapid growth of founder populations.

**Electronic supplementary material:**

The online version of this article (doi:10.1007/s10493-014-9856-1) contains supplementary material, which is available to authorized users.

## Introduction

Feather mites (Actinotrichida; Analgoidea and Pterolichoidea) are a group of over 2,400 named species (from a total of 12,000–16,000 probable species) of highly specialized plumage and skin ectocommensals present on every recent bird order, including the penguins (Gaud and Atyeo 1996; Proctor and Owens 2000; Proctor 2003; Dabert 2005; Mironov and Proctor 2008).

Feather mites are variously adapted for surviving in specific microhabitats on a bird’s body, i.e. down feathers, the vane surface of contour feathers, the interior of the quills or shafts of flight and tail feathers, and the surface of the skin or subcutaneous layers (Dabert and Mironov 1999; Proctor 2003). The mites inhabiting vanes are the most common and also most varied in body shape among feather mites. They display complex morphological adaptations to living in strong air-flows and the incessant movement caused by reciprocal friction of feathers during flight. The most common morphological adaptations include a strongly dorso-ventrally flattened and sclerotized body, and well developed membranous foot discs (ambulacra) that act as hold-fast organs (Dabert and Mironov 1999). These mites live predominantly on the ventral surface of the contour feathers (usually flight feathers) in narrow corridors between barbs.

It is generally agreed that feather mites complete their entire life cycle on the host’s body. The cycle involves four developmental stages after eclosion: six-legged larva, two nymphal instars (proto- and tritonymph), and adult. Although all the developmental stages are motile, there are almost no reports of feather mites from nesting material. Females and tritonymphs are thought to be the main dispersal stage (Mironov 2000). They invade new hosts almost exclusively through direct (feather-to-feather) physical contact between conspecific bird individuals: vertically from parents to offspring and horizontally during copulation or social behavior like grooming, communal roosting or aggression.

The host-ectocommensal relationships between feather mites and birds have been studied in two aspects: the possible influence of mites on host condition and cophylogenetic relationships to reveal the historical evolutionary events that created the recent bird-mite associations. The first group of studies is mainly based on correlative approaches, with the exception of Pap et al. (2005), and concerns the vane inhabiting mites of passerine birds. Results of these studies suggest rather weak effects of the presence of feather mites on the body condition of birds (for review see Galván et al. 2012). Cophylogenetic analyses mostly support cospeciation as the key factor explaining the observed host-ectocommensal associations; however, the other cophylogenetic events, like duplication, sorting, and host switching, may greatly complicate the pattern of the host-ectocommensal relationships (Dabert et al. 2001; Ehrnsberger et al. 2001; Dabert 2005).

As a consequence of their biology, feather mites are in general host-specific. A particular feather mite species usually inhabits a single bird species (monoxenous ectocommensal) or, less frequently, a few or several bird species (oligoxenous or polyxenous ectocommensal, respectively). Analyses based on morphological characters indicate that the multihost feather mites mostly are associated with closely-related birds (stenoxenous ectocommensals); however, there are examples of species that are present on a wide range of unrelated birds (euryxenous ectocommensals) (Euzet and Combes 1980; Dabert and Mironov 1999; Proctor 2003). Recent molecular phylogenetic analyses have revealed that some apparently oligoxenous feather mite species are in fact monoxenous cryptic species with little morphological differentiation (Whiteman et al. 2006; Badek et al. 2008; Dabert et al. 2008), or are composed of populations with levels of genetic differentiation suggesting early stages of speciation (Dabert et al. 2005).

**Fig. 1:**
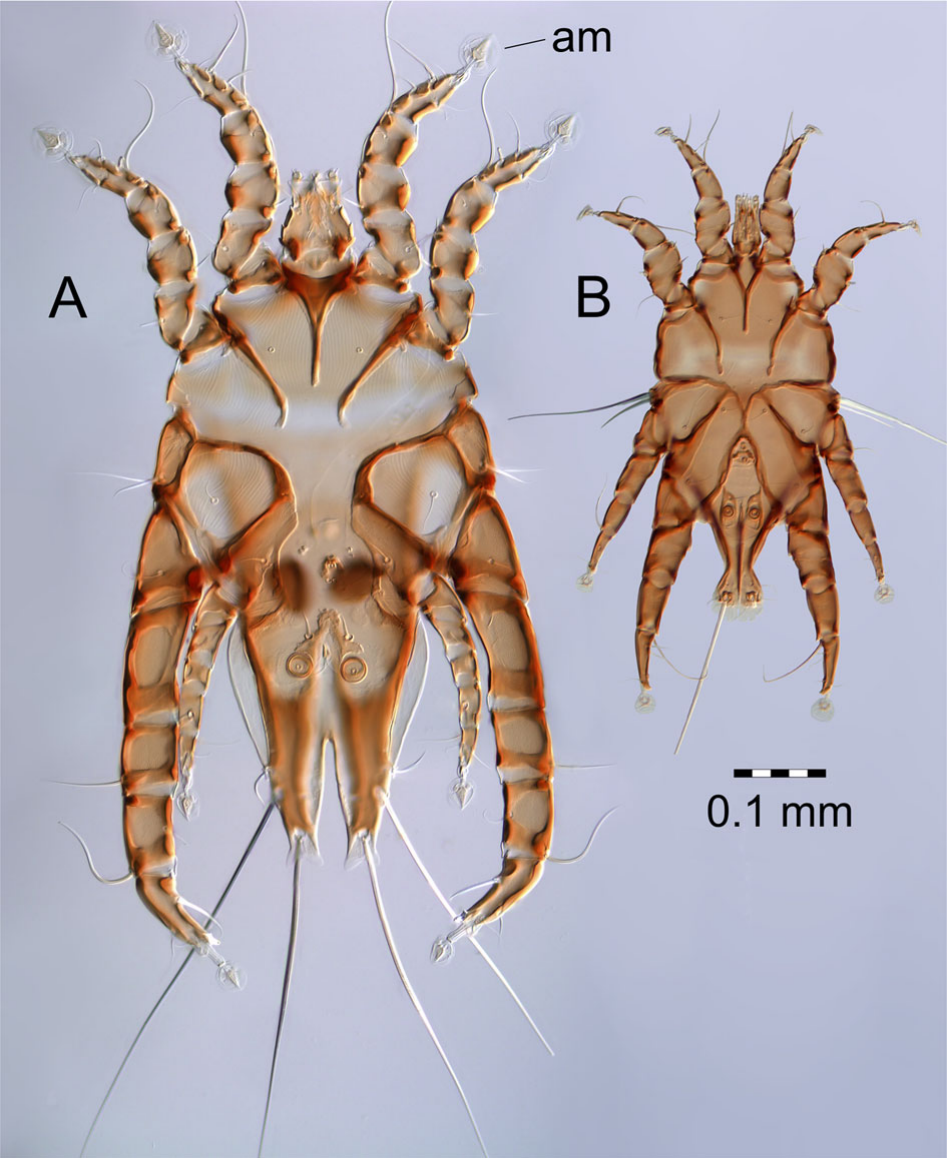
Males of *Zachvatkinia isolata* (*A*) and *Alloptes* (*Sternalloptes*) *stercorarii* (*B*) shown in the same scale, ventral view, am—ambulacrum

In this study we analyzed the DNA-barcode sequences of two feather mite species living on two sister species of birds. As a model we have chosen: arctic skua *Stercorarius parasiticus* L. and long-tailed skua *Stercorarius longicaudus* Vieillot breeding on tundra in the High Arctic archipelago of Svalbard and their two feather mite species *Zachvatkinia isolata* Mironov (Analgoidea, Avenzoariidae) and *Alloptes* (*Sternalloptes*) *stercorarii* (Dubinin) (Analgoidea, Alloptidae) which inhabit different parts of the plumage. The larger-bodied *Zachvatkinia* prefers wide corridors between barbs of the most exposed wing primaries, while smaller-bodied *Alloptes* inhabits most often secondaries and wing coverts (Fig. 1).

The sister relationship between arctic skua and long-tailed skua has been well established (e.g. Andersson 1999; Tavares and Baker 2008). Given that there are no reports about hybridization events between the arctic and long-tailed skuas (McCarthy 2006), we expected that both bird species would have a host-specific acarofauna. In the study area, the two host-bird species breed sympatrically, nests being 300–1,500 m apart. They defend their breeding territories against conspecifics and individuals of the other skua species in spectacular chase flights and direct physical contact may occur. If this contact is sufficient to transfer feather mites, we hypothesize that this would facilitate accidental contamination of the acarofauna resulting from interspecific aggressive behavior of the hosts. By analyzing DNA sequences of the cytochrome *c* oxidase subunit I (COI) and the hypervariable D2 region of large subunit ribosomal RNA gene (28S rDNA) from feather mite specimens sampled from arctic and long-tailed skuas, we were able to examine the degree of isolation of ectocommensal populations inhabiting these two host species.

## Materials and methods

### Study area

The Svalbard archipelago lies in the Norwegian Arctic between latitudes 74°N and 81°N and longitudes 10°E and 35°E, approximately 700 km north of mainland Norway. The land area of the archipelago amounts to 63,000 km^2^, of which 60 % is under permanent ice and snow. For the latitude the climate is relatively mild owing to the northern branch of the north Atlantic drift transporting heat northwards from lower latitudes. However, the long term annual mean temperature (1981–2010) at Ny-Ålesund is −5.2 °C (Førland et al. 2011) with only the four summer months, June to September, recording positive monthly averages peaking at +5.5 °C in July (Norwegian Meteorological Institute 2013).

### Sampled species

Birds were captured in Kongsfjord, Svalbard (78°N, 12°E) using a nest-trap or a netgun during the breeding season in June and July, 2010 and 2011. In total, we analyzed feather samples from 25 arctic skuas and 12 long-tailed skuas. The samples consisted of four to five barbs cut in vivo from the second primary flight feathers and preserved in 96 % alcohol. We chose feathers where *Zachvatkinia* is most abundant and *Alloptes*, quite rare even in its preferred habitat, is also possible to be found. Major wing coverts, that are the optimal microhabitat for *Alloptes,* are free of *Zachvatkinia* (Mironov 1981; Vasyukova and Mironov 1991). The collected samples contained from one to about 50 mite individuals per sample. In case of highly infested birds a maximum of five conspecific mites were sampled for molecular study. Four arctic skuas sampled in 2010 and one long-tailed skua sampled in 2011 were excluded from molecular analyses due to empty or small samples which failed in DNA extraction, resulting in a final host sample size of 21 arctic and 11 long-tailed skuas. For details concerning birds and feather mites used in the molecular study see Online Resource supplementary material, Tables A1 to A4.

Feather mite species were morphologically identified using Dubinin (1952), Mironov (1989) and Vasyukova and Mironov (1991). All mites found on the sampled feathers, including the chitinous exoskeletons remaining after DNA extraction, were used for analysis of acarofauna composition and morphospecies determination. Latin and English names of birds follow Dickinson (2003). Vouchers of feather mites (microslides) are deposited at the Department of Arctic Biology, University Centre in Svalbard (UNIS) and at the Department of Animal Morphology, Adam Mickiewicz University, Poznan, Poland.

**Fig. 2:**
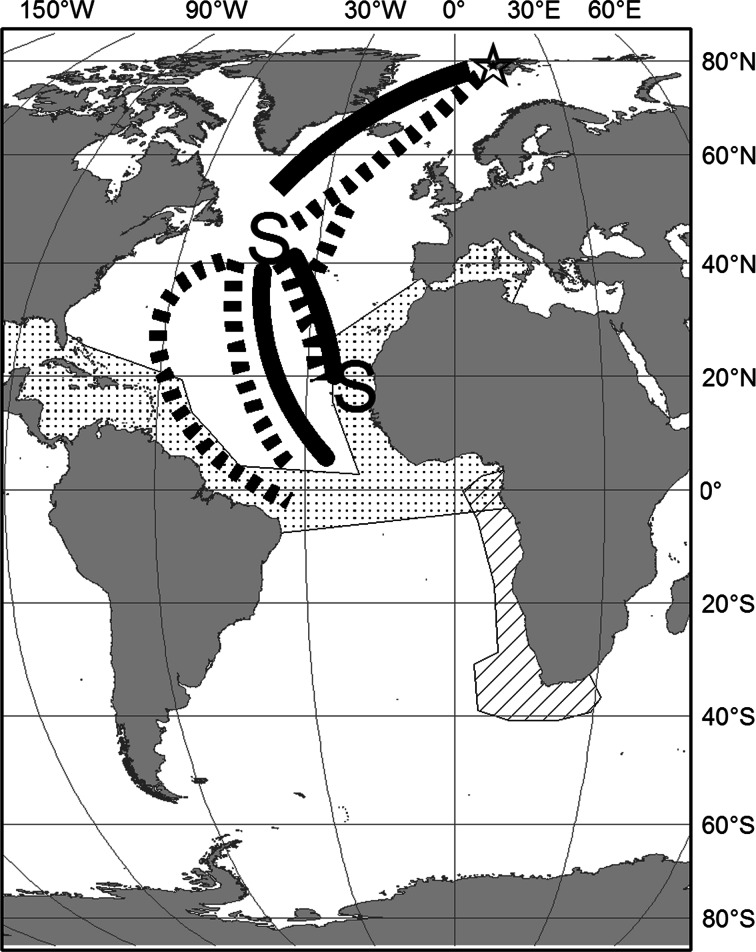
Stylized map of wintering areas of arctic skuas (*dotted area*) and long-tailed skuas (*hatched area*) from Svalbard, along with shared flyways during autumn (*solid lines*) and spring (*stippled lines*) and shared staging areas (S) in autumn and spring

All birds were equipped with light-level geolocators (mk13, mk15 or mk18 h, British Antarctic Survey, Cambridge, UK) attached to the leg band in order to track their inter-breeding distribution. Detailed information about the tracking data has been, or will be, published elsewhere (Gilg et al. 2013, unpublished data). Here, we schematically present the wintering areas along with information on shared flyways and staging areas, which are relevant for when and where physical contact can occur between the two species during the inter-breeding season (Fig. 2).

### Molecular analyses

Total genomic DNA was extracted from the individual mite specimens using a nondestructive method as described by Dabert et al. (2008). A 670-bp fragment of the mitochondrial cytochrome *c* oxidase subunit I (COI) gene was amplified with primers bcdF05 (5′-TTTTCTACHAAYCATAAAGATATTGC-3′) and bcdR04 (5′-TATAAACYTCDGGATGNCCAAAAAA-3′). An 850-bp fragment of the 28S rDNA, including D2 region, was amplified with primers 28SF0001 (5′-ACCCVCYNAATTTAAGCATAT-3′) and 28SR0990 (5′-CCTTGGTCCGTGTTTCAAGAC-3′) (Mironov et al. 2012). PCRs were carried out in 10 µl reaction volumes containing 5 µl Type-it Microsatellite PCR Kit (Qiagen, Hilden, Germany), 0.5 µM each primer, and 4 µl of DNA template using a thermocycling profile of one cycle of 5 min at 95 °C followed by 35 steps of 30 s at 95 °C, 90 s at 50 °C, 1 min at 72 °C, with a final step of 5 min at 72 °C. After amplification, the PCR products were diluted with 10 µl of water, and 5 μl of the diluted PCR reaction was analyzed by electrophoresis on a 1 % agarose gel. Samples containing visible bands were directly sequenced in forward direction using 1 μl of the PCR reaction and 50 pmol of forward sequencing primer. The COI sequences deposited in GenBank as species barcodes were additionally sequenced in the opposite direction using bcdR04 primer. Amplicons of the 28S rDNA were sequenced in both directions using internal primers 28SF0440 (5′-ACAAGTACCGTGAGGGAAAGTTG-3′) (Sonnenberg et al. 2007) and 28SR1000 (5′-GTCCGTGTTTCAAGACGGGTC-3′) (developed in this study). Sequencing was performed with BigDye Terminator v3.1 on an ABI Prism 3130XL Analyzer (Life Technologies). Contigs were aligned and manually assembled in ChromasPro v. 1.32 (Technelysium) and GeneDoc v. 2.7.000 (Nicholas and Nicholas 1997).

### Phylogenetic analysis, species delimitation, and haplotype genealogy

In total, we sequenced COI gene fragments from 83 specimens of putative *Z. isolata* (27 inhabiting long-tailed skuas and 56 inhabiting arctic skuas) and nine specimens of putative *A. stercorarii* (seven from arctic skuas and two from long-tailed skuas). The COI final alignment comprised 93 sequences including *Freyana* *anatina* Koch, Pterolichoidea (GenBank acc. no. GQ864352) used as an outgroup taxon. After species determination, seven specimens of *Z. isolata* representing both skua hosts and representatives of *Zachvatkinia stercorarii* Dubinin, *A. stercorarii*, and *Alloptes* sp. n. were sequenced for 28S rDNA analysis. All sequences were published in GenBank under accession nos. KJ804194-203, KF018820-911 (for details see Tables A1 and A2).

Phylogenetic trees were reconstructed using maximum parsimony (MP) and maximum likelihood (ML) approaches as implemented in PAUP 4* (Swofford 2002) and Garli v.2.0 (Zwickl 2006), respectively. The MP analysis was performed on unordered characters using standard unweighted heuristic search strategy with tree-bisection-reconnection (TBR) branch swapping and the starting tree obtained via stepwise addition with ten random-addition replicates. ML analysis was conducted with 30 search replications and the two-rate codon-based model. Support for tree branches was calculated by the nonparametric bootstrap method (BS) (Felsenstein 1985) with 100 replicates.

Pairwise distance calculations between sequences were computed using the Kimura two parameter (K2P) distance model (Kimura 1980) for all codon positions with MEGA 5 (Tamura et al. 2011). We assumed the minimum species boundary for COI sequences as at least tenfold the intraspecific variability. Also we applied two probability measures of species distinctiveness, reciprocal monophyly *P*_AB_ (Rosenberg 2007) and Randomly Distinct *P*_RD_ (Rodrigo et al. 2008). These analyses were conducted in Geneious 6.1.6 species delimitation plugin (Masters et al. 2011).

The network analyses of *Z. isolata* haplotypes was conducted using a data set comprising all COI sequences of *Z. isolata* collected from both host species in both years. The haplotype genealogy was revealed by two different approaches: statistical parsimony networks (SP) (Templeton et al. 1992) with 95 % connection limit as implemented in TCS 1.21 software (Clement et al. 2000) and the median-joining (M-J) network approach (Bandelt et al. 1999) as implemented in Network 4.5.1.0p software (http://www.fluxus-technology.com). To delete the superfluous links and median vectors in the calculated M-J network we applied the maximum parsimony postprocessing (Polzin and Daneschmand 2003).

We applied standard measures of within and between population differentiation for *Z. isolata* COI haplotypes. The probability that two randomly chosen haplotypes are different (haplotype diversity, Hd) and the average number of nucleotide differences per site between two randomly chosen DNA sequences (nucleotide diversity, π) were estimated using DnaSP 5.10.01 (Librado and Rozas 2009). The observed Hd and π were compared using predicted nucleotide diversity (π_p_) based on the relationship proposed by Goodall-Copestake et al. (2012). Hierarchically nested fixation index (F_ST_) for haplotypes sampled from the same host bird and host species was determined by an analysis of molecular variance (AMOVA) as implemented in Arlequin 3.5.1.3 (Excoffier and Lischer 2010).

## Results

### Acarofauna composition

Morphological analysis of the feather mite specimens collected from the feathers of the arctic and long-tailed skuas revealed three morphospecies of two analgoid genera: *A.* *stercorarii*, *Z. isolata*, and *Z. stercorarii*. *Zachvatkinia isolata* was commonly present on both skua species (92 and 97 % of the arctic skua and long-tailed skua acarofauna, respectively). The second feather mite species, *Z. stercorarii*, was found in small numbers (<1 % within the total feather mites counted). We found this species on two individuals of arctic skua, one individual captured in 2010 (sample T14 g) and a second bird captured in 2011 (T02p). On the latter bird individual *Z. stercorarii* formed a mixed population with *Z. isolata*. *Alloptes* mites were much less frequent than *Z. isolata* and they constituted 3 and 7 % of acarofauna of the arctic and long-tailed skuas, respectively.

**Fig. 3:**
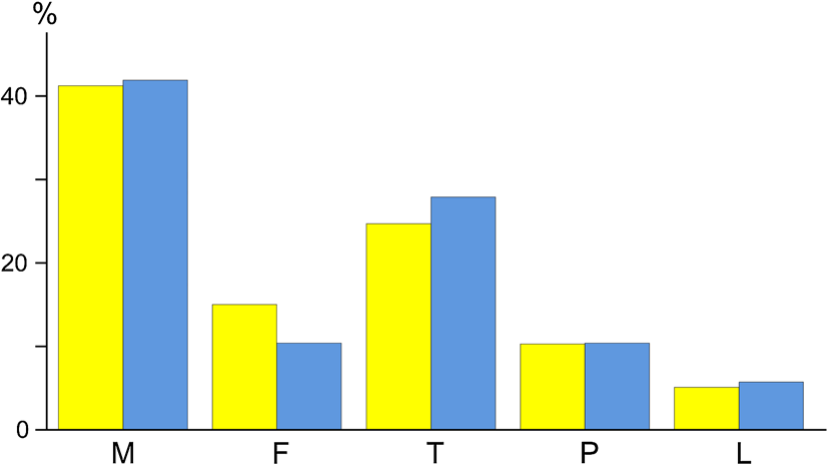
Composition of the *Zachvatkinia isolata* population on arctic skuas (*yellow*) and long-tailed skuas (*blue*) collected in Svalbard in 2010 and 2011. *M* male, *F* female, *T* tritonymph, *P* protonymph, *L* larva. (Color figure online)

The populations of *Z. isolata* inhabiting both bird host species displayed a very similar life stage composition (Fig. 3, for details concerning *Z.* *isolata* and *Alloptes* mites identified in each sample see Tables A3 and A4). The most common stage was the adult male (about 43 % in both hosts) while females were much less frequent (15.6 and 10.8 %, respectively). The combined contribution of summarized tritonymphs (coupled with males during precopulatory guarding) and adult females was roughly the same as for males (about 40 %). In *Alloptes* it was evident that males were extremely rare (6 %) and most of the population consisted of tritonymphs and females (78 % overall) (data not shown), but this observation should be treated cautiously because of a very small number of alloptid mites collected in this study.

### Phylogenetic trees

**Fig. 4:**
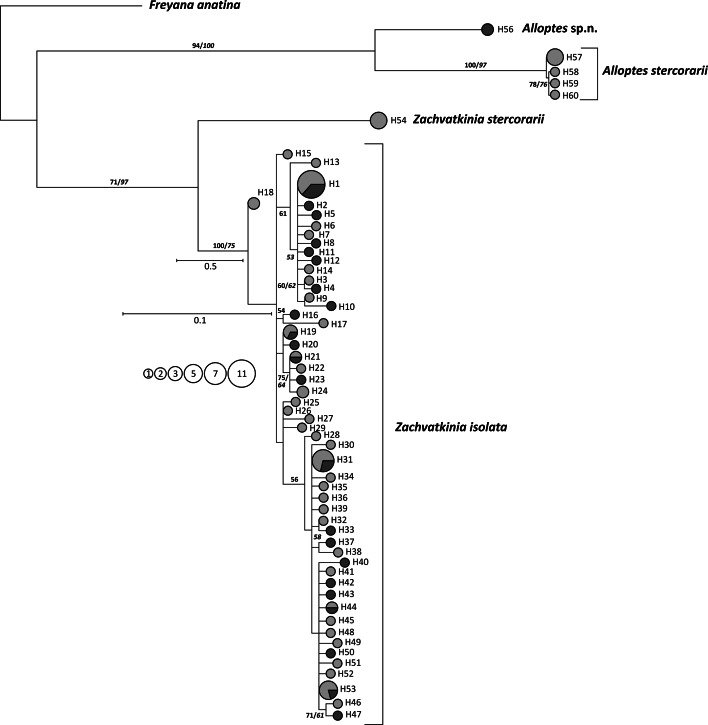
Maximum likelihood tree of the *Zachvatkinia* and *Alloptes* COI haplotypes found on arctic skuas (*yellow*) and long-tailed skuas (*blue*). Circle size is relative to the number of haplotype copies present in the dataset. Due to very short branches the part of the tree concerning *Z. isolata* is given in a larger scale (0.1). Outgroup haplotype—*Freyana anatina* (Pterolichoidea). The bootstrap support values >50 % are given at branches for MP (*regular*) and ML (*italic*). MP tree (not shown) was topologically very close to ML reconstruction. (Color figure online)

The maximum parsimony (MP) and maximum likelihood (ML) analyses of the COI haplotypes found in mites sampled from both skua species revealed the same pattern of relationships with two well supported clades corresponding to the feather mite genera *Alloptes* and *Zachvatkinia* (Fig. 4). The clade *Alloptes* included two stable subclades, one consisting of four haplotypes discovered in seven mite individuals sampled exclusively on the three arctic skuas, the second one restricted only to one haplotype found in two mite individuals inhabiting one specimen of the long-tailed skua. The subclades were tentatively named as *A. stercorarii* and *Alloptes* sp. n., respectively.

The *Zachvatkinia* clade also consisted of two subclades. The first subclade was restricted to *Z. stercorarii* and was represented by one COI haplotype found in mites collected on the arctic skuas. The second subclade grouped all COI haplotypes (N = 53) of *Z. isolata* found on both host species. *Zachvatkinia isolata* haplotypes did not display any host-dependent phylogenetic structure in MP and ML trees; the haplotypes originating from mites collected on both skua species were scattered within the clade with mostly poor support, numerous polytomies, and very short branches.

### Genetic distances and species delimitation

Although the COI sequences from *Z. isolata* inhabiting both skua hosts varied in 54 positions the average evolutionary distance (K2P) among them was relatively low and amounted to 1.07 % (SD 0.24) (Table 1). Almost all nucleotide substitutions were synonymous and did not affect amino acid sequence; however, two substitutions resulted in amino acid changes: the substitution of valine 51 with isoleucine in three mite specimens (Env417, Env566, Env578) collected on three different bird individuals, and the substitution of valine 96 with methionine in one mite specimen (Env536). Most of the observed substitutions were transitions which resulted in a relatively high average transition to transversion ratio (R = 7.64). *Zachvatkinia isolata* COI sequences treated as two different groups according to two different host species diverged by only 1.07 % (SD 0.24) and shared the same variable nucleotide positions. The probability measures of species delimitation have not been applied because of lack of support for the monophyly of the populations found on different hosts. Also the F_ST_ value for *Z. isolata* haplotypes sampled from arctic and long-tailed skuas was very low and amounted to <0.001 (SD 0.016; *P* = 0.49). *Zachvatkinia stercorarii* mites were sampled on two different arctic skuas captured in different years; however, they shared the same COI haplotype (H54). Two specimens of the new species *Alloptes* sp. n. also displayed one haplotype (H56) but they were sampled on the same bird (F02p). Among the seven *A. stercorarii* individuals analyzed, we found four different COI haplotypes which differed by 0.25 % (SD 0.13).

**Table 1:** Estimates of average evolutionary divergence (%) over COI sequence pairs between analyzed feather mite* Alloptes* and* Zachvatkinia* species

Population	Intraspecific	Interspecific				
		*Alloptes* sp. n.	*A. stercorarii*	*Z. isolata* SL	*Z. isolata* SP	*Z. stercorarii*
*Alloptes* sp. n.	n/c		1.94	2.26	2.27	2.32
*A. stercorarii*	0.25 (0.13)	19.39		2.17	2.17	2.16
*Z. isolata* SL	1.12 (0.26)	23.57	23.38		0.24	1.83
*Z. isolata* SP	1.02 (0.23)	23.69	23.44	1.07		1.84
*Z. stercorarii*	n/c	24.19	22.67	16.77	16.81	

Standard error estimates (in parentheses or above the diagonal) were obtained by a bootstrap procedure (500 replicates). Analyses were conducted using the Kimura 2-parameter method

*SL* mites sampled on long-tailed skuas, *SP* mites sampled on arctic skuas

Interspecific genetic distances among COI sequences from the four analyzed species ranged from about 17 % (between *Z. isolata* and *Z. stercorarii*) to 24.19 % (between *Z. stercorarii* and *Alloptes* sp. n.) (Table 1). The genetic distance between *A.* *stercorarii* from the arctic skua and *Alloptes* sp. n. sampled from long-tailed skua was 19.39 % (SD 1.94) and exceeded more than tenfold the intraspecific variability of *A. stercorarii* (0.25 %). Nucleotide substitutions observed in COI sequences from *Alloptes* sp. n. and *A. stercorarii* resulted in four amino acid changes (substitutions of glycine with serine, glutamic acid with aspartic acid, and isoleucine with valine in two sites). Moreover, both probability measures rejected the null hypothesis of random coalescent process (*P*_AB_ = 0.01, *P*_RD_ < 0.05) and the discovered distinct populations are most probably a pair of cryptic species. Similar support for species separation (*P*_AB_ < 0.01, *P*_RD_ < 0.05) was found between *Z. isolata* and *Z. stercorarii*, which are considered as separate species according to morphological characters.

The species status of the studied taxa revealed by COI sequence analyses was confirmed by the analysis of the nuclear DNA barcode. No intraspecific variability in the analyzed fragment of the 28S rDNA was detected in* A. stercorarii*. Mean genetic distance among 28S rDNA sequences of *Z. isolata* collected from arctic skuas and long-tailed skuas was similar to both intra-host values and amounted to 0.04 % (SD 0.04), while between *Z. isolata* and *Z. stercorarii* it amounted to 2.73 % (SD 0.54). A comparable genetic distance was observed between 28S rDNA sequences from *Alloptes* sp. n. and *A. stercorarii* (2.09 %, SD 0.51), which provides additional support for the cryptic speciation in alloptid mites inhabiting arctic skuas and long-tailed skuas.

### Genetic structure of haplotypes

**Fig. 5:**
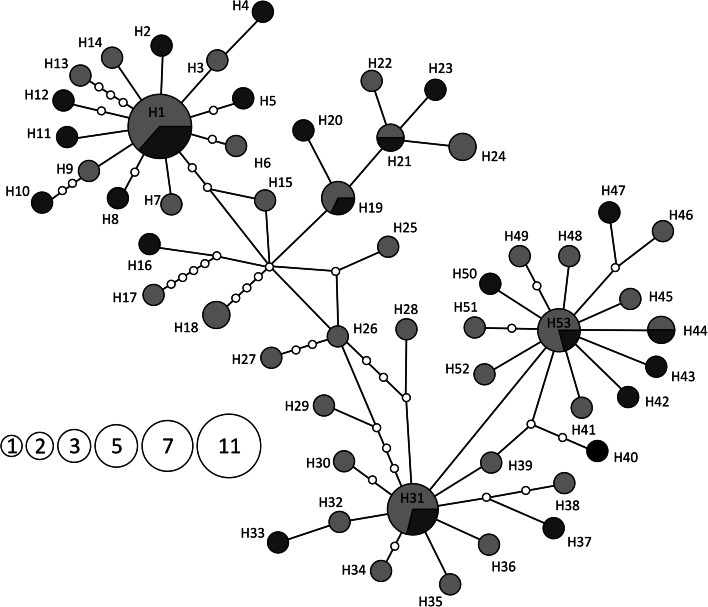
Median-joining haplotype networks showing genealogy of *Zachvatkinia isolata* haplotypes found on arctic skuas (*yellow*) and long-tailed skuas (*blue*). Circle size is relative to the number of haplotype copies present in the dataset. (Color figure online)

The networks of haplotype genealogies in *Z. isolata* were constructed using two different approaches (M-J and SP). Both methods produced congruent results which differed slightly in some intermediate connections (data not shown). Haplotypes of *Z. isolata* were not grouped according to host species but were distributed through the whole network (Fig. 5). Both network analyses revealed star-like structures with three high-frequency haplotypes (H1, H31, H53) surrounded by satellite haplotypes differing mainly by one to three substitutions from the central type. The central haplotypes H1 and H53 were the most distant from each other and a complex network of numerous intermediate haplotypes was reconstructed between haplotypes H1 and H31. Haplotypes were not grouped according to the mite hosts and the most common types (H1, H19, H21, H31, H44, and H53) were shared by mites inhabiting both skua species. The nucleotide diversity (π) values in *Z. isolata* populations were 0.01007 (SD 0.0006) and 0.01143 (SD 0.0007) for mites sampled from the arctic skua and long-tailed skua, respectively. The haplotype diversity (Hd) values for the same populations were 0.958 (SD 0.016) and 0.981 (both SD 0.019), respectively. Similar estimates of genetic diversity were obtained for all *Z. isolata* haplotypes collected from both skua species with Hd = 0.958 (SD 0.013) and π = 0.01039 (SD 0.0004). Predicted nucleotide diversity (π_p_) calculated from the observed haplotype diversity (Hd) for the same dataset was 0.00743 and deviated from the model expectation by 0.053. The nucleotide diversity for three star-like parts of the network showed much lower diversity than for the whole population (0.003, SD range 0.0006–0.0008 for each star) while the level of the haplotype diversity was high and amounted 0.783 (SD 0.093), 0.800 (SD 0.108), and 0.919 (SD 0.057) for H1, H31, and H53 stars, respectively.

## Discussion

The analysis of the plumage acarofauna of arctic and long-tailed skua yielded some new host-mite associations. Skuas are known to be hosts of two vane-inhabiting feather mite genera, *Alloptes* and *Zachvatkinia*, represented by three oligoxenous species: *A. stercorarii*, *Z. isolata*, and *Z. stercorarii*. So far, *A. stercorarii* has been reported from arctic skua, long-tailed skua, brown skua *S. antarcticus lonnbergi*, pomarine skua *S. pomarinus*, and south polar skua *S. maccormicki* (Dubinin 1952; Atyeo and Peterson 1967, 1970; Gaud 1976; Mironov 1989; Vasyukova and Mironov 1990, 1991). *Zachvatkinia isolata* is a typical feather mite for arctic skua and long-tailed skua (Mironov 1989; Vasyukova and Mironov 1990, 1991), while its possible sister species *Z. stercorarii* has been reported from the great skua *S. skua*, brown skua, pomarine skua, and south polar skua (Atyeo and Peterson 1967, 1970; Gaud 1976; Mironov 1989; Vasyukova and Mironov 1990, 1991). Here we report for the first time the presence of *Z. stercorarii* on arctic skua. Although this mite species was not common, it was found on two different bird individuals captured in two different years. This observation suggests the possibility of close physical contacts among arctic skua and other skua species. The *Z. stercorarii* individuals found on arctic skuas possibly have originated from the great skuas that also breed in Kongsfjorden, some of them close to arctic skua nests (<300 m), but never close to long-tailed skua nests (>1,500 m) (BM pers. obs.).

The second new host-mite association is the discovery of a new cryptic species of the *Alloptes* genus. Results of our molecular analyses revealed that the alloptid population from arctic skua is genetically differentiated at the species level. This observation was expected because recent analyses based on molecular data show that the populations of oligoxenous feather mite species inhabiting different host species usually turn out to be genetically isolated and often differentiated at the level of closely-related species (Dabert et al. 2005, 2008; Badek et al. 2008; Mironov et al. 2012).

In contrast to *Alloptes*, our results indicate that *Z. isolata* populations collected from arctic skuas and long-tailed skuas are conspecific and do not display a host-dependent genetic structure of population. *Zachvatkinia isolata* mites show the same age and sex structure on both host species which support hypothesis that they could constitute a single population. This observation was corroborated by molecular analyses. Neither the structure of the phylogenetic tree nor the genetic distance analyses point to the genetic distinctiveness of *Z. isolata* populations from arctic and long-tailed skuas. Also the very low F_ST_ value for mites inhabiting both skua species strongly suggests almost free interbreeding among the members of both populations and indicates the possibility of a very close physical contact among individuals of both bird species during the dispersal period of *Zachvatkinia* mites. In contrast, the *P*_AB_ and *P*_RD_ statistics and genetic distance values observed for *Z. isolata* and *Z. stercorarii* populations captured on the same host individuals support the specific status of both feather mite species, which are hard to discriminate by phenotypic features.

What could be a reason for this discrepancy in the genetic structure of *Alloptes* and *Zachvatkinia* populations? Phylogenetic reconstructions based on morphological characters suggest that the genus *Zachvatkinia* originated on procellariform birds and then shifted to the new host group, Charadriiformes, after differentiation of the ancestral charadriiomorph birds into Charadrii and Lari (Mironov 1991; Dabert and Mironov 1999). Mites of the genus *Zachvatkinia* are large, strongly sclerotized, and morphologically uniform and coexist on procellariiform birds with two other mite genera, *Rhinozachvatkinia* and *Promegninia*. Similar to *Alloptes*, mites belonging to the genera, *Promegninia* and *Rhinozachvatkinia* (Avenzoariidae) are small-bodied, weakly sclerotized, and show high interspecific morphological variation. Larger *Zachvatkinia* mites inhabit the vanes of large flight feathers while smaller *Promegninia* and *Rhinozachvatkinia* occur on smaller coverts of the wing. A horizontal transfer of the *Zachvatkinia* mites to charadriiform birds took place, but there are no representatives of *Promegninia* and *Rhinozachvatkinia* on these hosts. These mites probably did not colonize charadriiform birds because they inhabit the more protected and inaccessible parts of the plumage (Dabert and Mironov 1999), and hence are less likely to be transferred by casual contact than those living on the surfaces of flight feathers, like *Zachvatkinia*. Taking into account the different microhabitat preferences of mites, a similar explanation could also be valid for the *Zachvatkinia*-*Alloptes* associations with skuas. *Zachvatkinia* prefers the primary and secondary flight feathers (Mironov 1989) while *Alloptes* is mainly restricted to deeper parts of plumage (coverts) and only occasionally is found on flight feathers (Mironov 1981; Vasyukova and Mironov 1991). Unpublished data kindly provided us by P. W. Schaefer show that *Z. isolata* inhabits exclusively most outer primary flight feathers (1–8) while *A. stercorarii* is found evenly distributed on primary and secondary flight feathers with population maximum on wing coverts. Additionally, the same data of Schaefer confirm our observation that on the exposed primary flight feathers *Z. isolata* is several times more frequent than *A. stercorarii*. In our opinion it points to rather frequent but transient contacts between two skua species when the probability of the acarofauna exchange is much higher for large in numbers and inhabiting exposed parts of primary flight feathers *Zachvatkinia* than much more rare *Alloptes* living primarily in more protected and inaccessible parts of the plumage.

The effect of host behavior on ectoparasite population genetic structure has been studied in bats. The social interaction of the host outside of the maternity period, such as mating and hibernation, provides opportunities for extensive parasite exchange between *Myotis myotis* Borkhausen individuals and results in very low genetic differentiation and no evidence for population substructuring in *Spinturnix myoti* Kolenati mites (van Schaik et al. 2014). Physical contact between hosts is also postulated as a reason for lack of host-dependent genetic structure in the parasitic bat fly *Cyclopodia horsfieldi* Meijere (Diptera: Hippoboscoidea) collected from three bat species of *Pteropus* (Olival et al. 2013). A haplotype network pattern and parameters of genetic structure (very low F_ST_ and π values, high Hd value), very similar to *Z. isolata*, were explained as a consequence of frequent physical contacts between co-mingling flying fox species on sequentially used roosting sites and subsequent high level of parasite gene flow. The other possibility of parasite oligoxeny postulated by the authors was sequential use of a roosting site within a 2–3 week window where flies may emerge from metamorphosis in the roost substrate. However, this route is not possible in feather mites as they complete the entire life cycle on the bird’s body. Moreover, we exclude the possibility of *Zachvatkina* transfer via the soil in the common breeding area of arctic and long-tailed skuas. Feather mites do not survive longer than three to ten days off the host body and dispersal over the some hundred meters between breeding birds is unfeasible because they are too morphologically specialized to walk well on non-feather surfaces (Proctor and Owens 2000).

At present, the nature of contacts between skuas that enable free transmission routes for mites and the area(s) where this might take place (nesting area, migration, wintering area) are poorly understood. We think direct physical contact during the breeding season provides the most plausible mechanism, when the skuas may engage in air combats. This combat is best characterized as an aerial chase occurring at high speed, and we only see them briefly touching each other. However, this contact may be sufficient to enable transfer of mobile feather mites. The breeding population of long-tailed skua is small in Svalbard, i.e. <50 pairs (Kovacs and Lydersen 2006). In the study area (Kongsfjorden), they breed in a restricted area of <15 km^2^, and physical contact between long-tailed skuas and arctic skuas is probably restricted to this particular area, when arctic skuas enter the territory of long-tailed skuas. Further transfer of mites would be possible both horizontally and vertically within each species via intra-specific physical contact (such as intraspecific territorial air combat) and transfer to offspring, respectively.

During the inter-breeding season host birds migrate over vast distances along the Atlantic Ocean (Fig. 2), and the potential for interspecific physical contact is much lower. They do, however, partly overlap in their distributions, but it is less likely that they engage in air combat when they have no nesting territory to defend. The potential for contact is greatest during spring, when they share common staging areas in West Africa, the North Atlantic (between Newfoundland and the Azores) and along flyways (Fig. 2). They also share flyways during autumn, but the long-tailed skuas migrate earlier than the arctic skuas and the temporal overlap is very low. During winter they are spatially segregated, with only some overlap in the Gulf of Guinea (see Fig. 2).

The genetic structure of the *Z. isolata* population points to some aspects related to dispersal of the feather mites. In haplotype networks, structures with a dominant central haplotype surrounded by several satellite haplotypes are considered to represent the recent origin and rapid subsequent population expansion (Ferreri et al. 2011; de Jong et al. 2011). It is also assumed that population size expansion follows a former bottleneck or founder event (Slatkin and Hudson 1991; Nyström et al. 2010). In a genetic context these structures are characterized by high haplotype diversity (Hd) and low nucleotide diversity (π) as is observed in *Z. isolata.* The conception of founder effect and subsequent rapid population growth is consistent with our knowledge about the dispersal strategy of most species of feather mites, including *Zachvatkinia*. Individual birds are infected vertically (usually from parents to young birds or during copulation) by some fertilized mite females and tritonymphs (Dabert and Mironov 1999; Mironov 2000); however, our observations suggest that horizontal transmission may be quite frequent as well.

*Zachvatkinia isolata* mites appear to form a highly genetically diverse and large population on the studied skua species. Although our analyses concerned only a relatively small subsample of this large population, our estimates of genetic diversity in *Z. isolata* population fit well to the 75–95 % in the Goodall-Copestake et al. (2012) model expectations with the deviation value of ca. 0.05. This result suggests that the sample size do not biased the results. However, our observations concerning the genetic structure of *Z. isolata* population should be tested by further investigations based on more numerous mites sampled also from recaptured birds.

### Data accessibility

We provide Online Resource supplementary material (Tables A1 to A4) containing detailed information about sampled birds, mites, and DNA sequences used to perform all presented analyses. The sequences were deposited in GenBank under accession numbers KF018820-KF018911 (COI) and KJ804194-KJ804203 (28S rDNA).

## Electronic supplementary material

Below is the link to the electronic supplementary material.

Supplementary material 1 (PDF 103 kb)

Supplementary material 2 (PDF 89 kb)

Supplementary material 3 (PDF 95 kb)

Supplementary material 4 (PDF 96 kb)
