# Supplementary material for: Differences in speciation progress in feather mites (Analgoidea) inhabiting the same host: the case of Zachvatkinia and Alloptes living on arctic and long-tailed skuas
Source: Exp Appl Acarol. 2014 Oct 24;65(2):163–79. doi: 10.1007/s10493-014-9856-1 (PMC4274374; doi:10.1007/s10493-014-9856-1)
Supplement: Supplementary file 1 — Supplementary material 1 (PDF 103 kb) [file 10493_2014_9856_MOESM1_ESM.pdf]

Table A1. Feather mites (FM) sampled from arctic skua used for molecular study.

Sample ID	FM specimen & DNA code	COI haplotype	GenBank Acc. nos.		FM species
			COI	28S rDNA	
T01p	Env481	H18	KF018867		*Zachvatkinia isolata*
	Env482	H30	KF018868		*Z. isolata*
	Env484	H18	KF018869		*Z. isolata*
	Env485	H1	KF018870		*Z. isolata*
	Env480	H59	KF018860		*Alloptes stercorarii*
T02p	Env478	H31	KF018866		*Z. isolata*
	Env479	H54	KF018820	KJ804194	*Z. stercorarii*
T03p	Env470	H58	KF018830		*A. stercorarii*
	Env471	H57	KF018831		*A. stercorarii*
	Env472	H57	KF018832		*A. stercorarii*
	Env473	H57	KF018833	KJ804203	*A. stercorarii*
T04p	Env546	H31	KF018827		*Z. isolata*
	Env547	H34	KF018862		*Z. isolata*
	Env548	H29	KF018863		*Z. isolata*
	Env549	H53	KF018864	KJ804196	*Z. isolata*
T05p	Env574	H25	KF018906	KJ804195	*Z. isolata*
	Env576	H39	KF018907		*Z. isolata*
T06g	lack of sequence data				
T06p	Env551	H31	KF018890		*Z. isolata*
	Env552	H13	KF018891		*Z. isolata*
	Env553	H28	KF018892		*Z. isolata*
	Env554	H1	KF018893		*Z. isolata*
T07g	lack of sequence data				
T07p	Env441	H52	KF018861		*Z. isolata*
T08g	empty sample				
T08p	Env536	H7	KF018881		*Z. isolata*
	Env538	H21	KF018882		*Z. isolata*
	Env539	H49	KF018883		*Z. isolata*
	Env540	H17	KF018884		*Z. isolata*
	Env469	H57	KF018859		*A. stercorarii*
	Env583	H60	KF018829		*A. stercorarii*
T09g	Env532	H1	KF018879		*Z. isolata*
	Env533	H53	KF018880		*Z. isolata*
T09p	Env556	H9	KF018894		*Z. isolata*
	Env557	H19	KF018895		*Z. isolata*
T10g	Env524	H51	KF018876		*Z. isolata*
	Env528	H48	KF018877		*Z. isolata*
T10p	Env570	H32	KF018902		*Z. isolata*
	Env571	H1	KF018903		*Z. isolata*
	Env572	H24	KF018904	KJ804201	*Z. isolata*
	Env573	H24	KF018905		*Z. isolata*
T11g	lack of sequence data				
T11p	Env560	H31	KF018896		*Z. isolata*
	Env562	H26	KF018897		*Z. isolata*
	Env564	H35	KF018898		*Z. isolata*
	Env565	H38	KF018899		*Z. isolata*
T12g	Env520	H53	KF018872		*Z. isolata*
	Env521	H31	KF018873		*Z. isolata*

	Env522	H46	KF018874		*Z. isolata*
	Env523	H19	KF018875		*Z. isolata*
T12p	Env566	H44	KF018900		*Z. isolata*
	Env569	H53	KF018901	KJ804197	*Z. isolata*
T13g	Env447	H45	KF018887		*Z. isolata*
	Env448	H15	KF018888		*Z. isolata*
	Env449	H1	KF018889		*Z. isolata*
	Env450	H3	KF018865		*Z. isolata*
T13p	Env577	H27	KF018908		*Z. isolata*
	Env578	H22	KF018909		*Z. isolata*
	Env579	H6	KF018910		*Z. isolata*
	Env580	H1	KF018911		*Z. isolata*
T14g	Env529	H54	KF018821		*Z. stercorarii*
	Env530	H54	KF018822		*Z. stercorarii*
	Env531	H54	KF018878		*Z. stercorarii*
T14p	Env541	H36	KF018885		*Z. isolata*
	Env544	H41	KF018886		*Z. isolata*
T15g	Env412	H14	KF018828		*Z. isolata*
T16g	Env517	H1	KF018871		*Z. isolata*
