# Supplementary material for: Differences in speciation progress in feather mites (Analgoidea) inhabiting the same host: the case of Zachvatkinia and Alloptes living on arctic and long-tailed skuas
Source: Exp Appl Acarol. 2014 Oct 24;65(2):163–79. doi: 10.1007/s10493-014-9856-1 (PMC4274374; doi:10.1007/s10493-014-9856-1)
Supplement: Supplementary file 2 — Supplementary material 2 (PDF 89 kb) [file 10493_2014_9856_MOESM2_ESM.pdf]

Table A2. Feather mites (FM) sampled from long-tailed skua used for molecular study.

Sample ID	FM specimen & DNA code	COI haplotype	GenBank acc. nos.		FM species
			COI	28S rDNA	
F01p	Env491	H10	KF018841		*Zachvatkinia isolata*
F02p	Env417	H44	KF018836		*Z. isolata*
	Env418	H43	KF018837		*Z. isolata*
	Env420	H37	KF018838		*Z. isolata*
	Env421	H21	KF018839		*Z. isolata*
	Env487	H56	KF018834		*Alloptes* sp.n.
	Env488	H56	KF018835	KJ804202	*Alloptes* sp.n.
F03p	lack of sequence data				
F04p	Env504	H12	KF018849		*Z. isolata*
	Env505	H2	KF018850		*Z. isolata*
	Env506	H31	KF018851		*Z. isolata*
	Env507	H16	KF018852	KJ804199	*Z. isolata*
	Env508	H53	KF018853		*Z. isolata*
F05p	Env502	H4	KF018847		*Z. isolata*
	Env503	H1	KF018848		*Z. isolata*
F07p	Env495	H23	KF018842	KJ804200	*Z. isolata*
F08p	Env451	H11	KF018840		*Z. isolata*
F09p	Env497	H42	KF018843		*Z. isolata*
	Env498	H31	KF018844		*Z. isolata*
	Env499	H1	KF018825	KJ804198	*Z. isolata*
	Env500	H1	KF018845		*Z. isolata*
	Env501	H40	KF018846		*Z. isolata*
FJ03g	Env509	H33	KF018854		*Z. isolata*
	Env510	H20	KF018855		*Z. isolata*
FJ04g	Env425	H47	KF018826		*Z. isolata*
FJ05g	Env512	H5	KF018856		*Z. isolata*
	Env513	H1	KF018823		*Z. isolata*
	Env514	H19	KF018857		*Z. isolata*
	Env515	H50	KF018858		*Z. isolata*
FJ06g	Env516	H8	KF018824		*Z. isolata*
