# Supplementary material for: Differences in speciation progress in feather mites (Analgoidea) inhabiting the same host: the case of Zachvatkinia and Alloptes living on arctic and long-tailed skuas
Source: Exp Appl Acarol. 2014 Oct 24;65(2):163–79. doi: 10.1007/s10493-014-9856-1 (PMC4274374; doi:10.1007/s10493-014-9856-1)
Supplement: Supplementary file 3 — Supplementary material 3 (PDF 95 kb) [file 10493_2014_9856_MOESM3_ESM.pdf]

Table A3. ***Zachvatkinia isolata*** **and** ***Alloptes stercorarii*** **mites collected from the feathers of arctic skuas. M – male, F – female, T – tritonymph, P – protonymph, L – larva.**

		Zachvatkinia isolata					*Alloptes stercorarii*				
**Sample ID**	**Collection date**	**M**	**F**	**T**	**P**	**L**	**M**	**F**	**T**	**P**	**L**
T06g	July 2010	13	5	13	0	0	0	0	0	0	0
T07g	July 2010	2	1	1	0	1	0	0	0	0	0
T08g	July 2010	0	0	0	0	0	0	11	2	0	1
T09g	July 2010	3	5	0	2	1	0	0	0	0	0
T10g	July 2010	13	2	6	5	2	0	0	0	0	0
T11g	July 2010	2	0	2	0	0	0	0	0	0	3
T12g	July 2010	10	9	6	10	3	0	0	0	0	0
T13g	July 2010	5	3	2	2	1	0	0	0	0	0
T14g	July 2010	2	0	1	2	0	0	0	0	0	0
T15g	July 2010	7	3	1	0	0	0	0	0	0	0
T16g	July 2010	1	1	1	0	0	0	0	0	0	0
T1p	30 June 2011	3	1	0	0	0	1	0	0	0	0
T2p	30 June 2011	2	0	1	0	0	0	1	0	0	0
T3p	30 June 2011	0	0	0	0	0	1	6	1	1	0
T4p	30 June 2011	5	1	6	0	0	0	0	0	0	0
T5p	2 July 2011	10	0	5	0	1	0	0	0	0	0
T6p	3 July 2011	13	1	5	0	0	0	0	0	0	0
T7p	4 July 2011	2	2	1	0	0	0	0	0	0	0
T8p	4 July 2011	14	5	7	8	1	0	0	0	0	0
T9p	6 July 2011	16	5	4	3	4	0	0	0	0	0
T10	6 July 2011	6	0	4	0	0	0	0	0	0	0
T11p	6 July 2011	23	6	12	5	6	0	0	0	0	0
T12p	6 July 2011	8	3	13	5	2	0	0	0	0	0
T13p	7 July 2011	15	10	12	4	0	0	0	0	0	0
T14p	7 July 2011	3	1	3	0	0	0	0	0	0	0
	Total:	178	64	106	46	22	2	18	3	1	4
